# 

**Published:** 1885-04

**Authors:** 


### THE MEDICAL ASSOCIATION OF GEORGIA.

The Medical Association of Georgia will convene in Savannah April 15th, 1885, at 11 o'clock a. m., at Metropolitan Hall.

### PROGRAMME.

The exercises will open with prayer by the Rev. Charles H. Strong, when an address of welcome will be extended to the Association by Dr. William H. Elliott, President of the Georgia

Medical Society, and also by Hon. Rufus E. Lester, Mayor of the city. Arrangements have been made for the annual oration, to be delivered on the evening of the same day, Dr. Howard J. Williams, of Macon, orator.

Thursday, at 1 o'clock p. m., an invitation will be extended to the Association to participate in an excursion to Tybee Island, visiting, also, other points of interest.

The charges at the several hotels have been fixed as follows: Pulaski House $3.00 per diem; Screven House $3.00 per diem; Marshall House $2.00 per diem; Harnett House $2.00 per diem.

The Georgia Medical Society will endeavor to render the meeting as pleasant and agreeable as possible to the members, and earnestly hope for a large attendance.

Committee of Arrangements—Drs. W. Duncan, J. B. Read, J. C. LeHardy, R. J. Nunn, George H. Stone, T. J. Charlton, W. H. Elliott, R. B. Harris, J. M. Johnston, C. H. Colding.

